# Aging Dogs Manifest Myopia as Measured by Autorefractor

**DOI:** 10.1371/journal.pone.0148436

**Published:** 2016-02-10

**Authors:** Jerome Hernandez, Cecil Moore, Xuemei Si, Stuart Richer, Janet Jackson, Wei Wang

**Affiliations:** 1 Nestlé Purina Research, St Joseph, Missouri, United States of America; 2 Department of Veterinary Medicine and Surgery, University of Missouri, Columbia, Missouri, United States of America; 3 Nestlé Research Center, St Louis, Missouri, United States of America; 4 Ophthalmology, Captain James A Lovell Federal Health Care Facility, North Chicago, Illinois, United States of America; Sun Yat-sen University, CHINA

## Abstract

**Objective:**

Dogs, like humans, experience eye changes with aging: hardening and clouding of the lens and accumulated oxidative damage from UV sunlight. It has been debated whether such changes could be affecting the visual function of dogs. The objective of this study was to determine if autorefractometry could be used to measure visual function in dogs.

**Animals and Methods:**

Nine Beagle dogs (ages 1 to 14 years) were examined by a veterinary ophthalmologist and their eyes determined to be free of cataracts. Spherical equivalent refractive error was measured by handheld autorefractor (Welch Allyn SureSight) under both indirect and direct lighting conditions with five measurements per condition, per eye. Measures were repeated on three different days for each dog within six weeks. Nonparametric statistics were used to detect differences among lighting conditions and test days, and between eyes. Spearmen correlation assessed the visual measurement outcomes’ association with age.

**Results:**

There was no difference for day-to-day or between-eye measurements. Significantly, the Beagles showed a myopic shift with aging (average spherical equivalent ranged from plano to -3.00 diopters), suggesting that dogs become more near-sighted as they age (r = -0.48 and -0.73 under direct and indirect lights; p<0.05 both). Younger dogs were able to make larger accommodation changes from indirect light to direct light conditions, indicating a more flexible lens (r = -0.50, p<0.05).

**Conclusions:**

Although designed for humans, the hand-held autorefractor technique is applicable to dogs and sensitive to light conditions. The age-associated myopic shift could be expected to compromise dogs’ visual functions.

## Introduction

Eyesight is one of the key senses for acquiring information from the outside world. In the wild, dogs depend on keen eyesight for hunting, which is essential for their daily survival. Domesticated dogs need this key sense to interact with their environment, for visual cues from their owners and for communications with humans and other animals.

Dogs, like humans, experience eye changes with aging, i.e., hardening and clouding of the lens and accumulated oxidative damage from UV sunlight. Development of cloudy lenses in older dogs, referred to as nuclear sclerosis, occurs with the aging process [1]. The cloudy lens of older dogs is readily visible to the naked eye as an observed hazy or bluish appearance within the pupil space. This is often viewed by owners as suspected cataract formation, and is one of the leading concerns for owners presenting their dog to veterinarians for ocular examination [2].

In veterinary ophthalmology, nuclear sclerosis in older dogs is considered to occur as part of normal aging change and is believed to result from internal compression and an increased density of the lens nucleus [3]. It is not generally believed to significantly affect vision in dogs, except in unusually dense or advanced cases. However, the clinical distinction between advanced nuclear sclerosis and early nuclear senile cataract in dogs is often indistinct [3]. In contrast, similar changes in older humans are considered a type of cataract which may be associated with lens nuclear brunescence and is referred to as nuclear cataract or senile cataract. These cataracts contribute to refractive error shift towards nearsightedness [4,5], and can be a significant source of visual impairment in humans [6].

Objective refractive evaluation methods are available for human visual function assessment. One method that may be applicable for dogs uses a fast 5-second autorefractor for testing refractive errors in infants and toddlers who cannot yet communicate well. The portable handheld autorefractor has light and sound that engage test subjects’ attention, with minimal cooperation required [7,8].

The objective of this study was to evaluate this refractor technique for use in dogs, and to determine if the autorefractor could be used to measure visual impairment, if any, in dogs of different ages, and to determine if such a method is repeatable and sensitive enough to be useful in detecting visual function changes.

## Methods

### Animals

Nine adult Beagles ages 1 to 14 years were selected, representing different age groups, at one of our pet centers. All dogs were in good health and had normal eyes as determined by a complete eye examination performed by a board certified veterinary ophthalmologist (CM). Any evidence of cataracts or nuclear sclerosis was noted (Table 1). Throughout the study, the dogs were housed in pairs, with continuous free access to be indoor or outdoor, and were provided with opportunities for outdoor exercise and social interactions. All dogs were fed individually to maintain body weight and were provided with water ad libitum. The dogs were monitored daily by veterinary and care staff. The dogs were selected from a closed research colony where they spend their entire lives and were returned to the colony at the end of study. This study protocol was reviewed and approved by the Nestlé Purina Animal Care and Use Committee.

**Table 1:** Characteristics of 9 Beagles.

Dog #	Age (years)	Gender	Nuclear Sclerosis (presence or not)	Nuclear Sclerosis Severity (early, mild, moderate or dense)
1	1.17	F	No	
2	1.19	M	No	
3	3.19	F/s	No	
4	3.19	M/n	No	
5	5.93	F/s	No	
6	5.93	M/n	No	
7	8.88	F/s	Yes	mild
8	10.22	M/n	Yes	mild/moderate
9	13.65	M/n	Yes	dense

F: Female; F/s: Female sprayed; M: Male; M/n: Male/neutered

Nuclear sclerosis presence or not and severity was determined by board certified veterinarian ophthalmologist. The severity degree was classified as: early, mild, moderate, or dense stage.

### Autorefraction procedure

Spherical equivalent refractive error was measured by a handheld autorefractor (Welch Allyn SureSight, Skaneateles Falls, NY, USA) on separate days from the initial screening evaluation. Autorefractor measurements were done under both indirect and direct lighting conditions with five measurements per condition, per eye. Measures were repeated on three different days for each dog within six weeks. The dogs’ eyes were not dilated for autorefractor tests.

The indirect lighting condition (illumination ~125 lux) was set with indoor light from an adjacent room coming through an open door into a dark room with dogs facing the incoming light. The direct light condition (illumination ~1,100 lux) was set in the same position but with the light turned on in the examination room. Eyes were first tested under the indirect lighting condition, followed by direct lighting at the same setting. The difference in refractive error as measured in diopter (D) from the indirect lighting condition to the direct lighting condition was taken as an indicator of accommodative capacity of the eyes.

Spherical equivalent was calculated by sphere + 0.5 cylinder. Results were presented as mean ± standard error of five measurements at each condition, per eye and per day.

### Statistics

Spearmen correlations were used to assess the visual measurement outcomes’ association with age. Linear mixed models were conducted to 1. Detect differences among lighting conditions, test days, and between eyes; and 2. Compare spherical equivalent refractive error among three measures on each day: 1st measure, Average 3 measure, or Average 5 measure. P value <0.05 was considered statistically significant (SAS 9.3 SAS Institute Inc., Cary, NC, USA).

## Results

**Fig 1:**
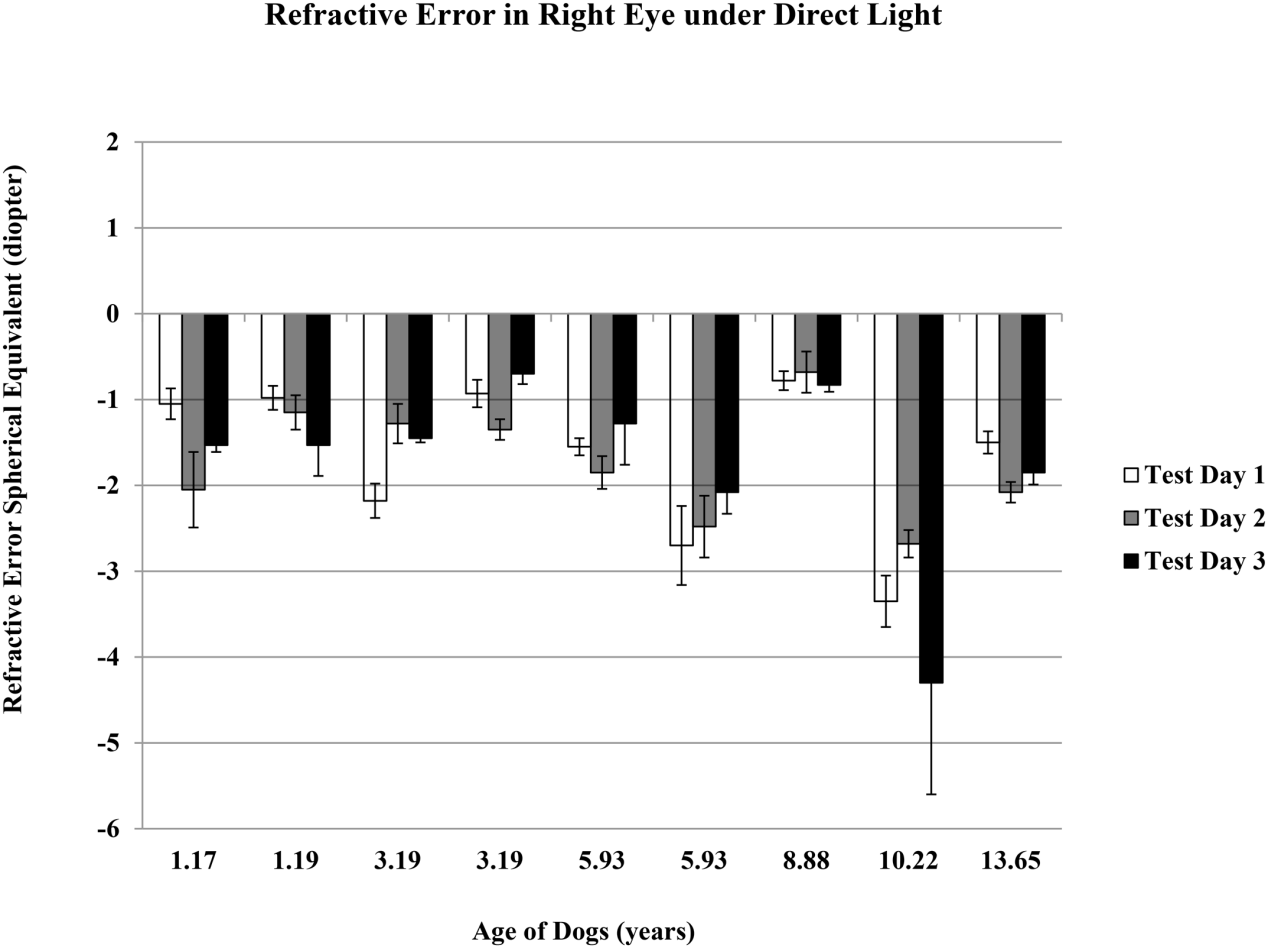
Refractive Error in Right Eyes under Direct Light. Refractive error spherical equivalent was the average of total five measurements determined using auto-refractor under the direct light condition of ~ 1,100 lux. Data was for right eye, and on three test days.

**Fig 2:**
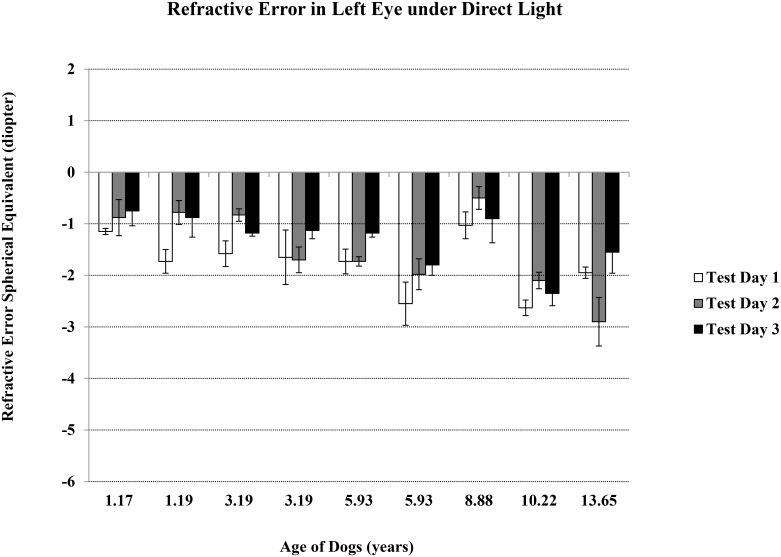
Refractive Error in Left Eyes under Direct Light. Refractive error spherical equivalent was the average of total five measurements determined using auto-refractor under the direct light condition of ~ 1,100 lux. Data was for left eye, and on three test days.

**Fig 3:**
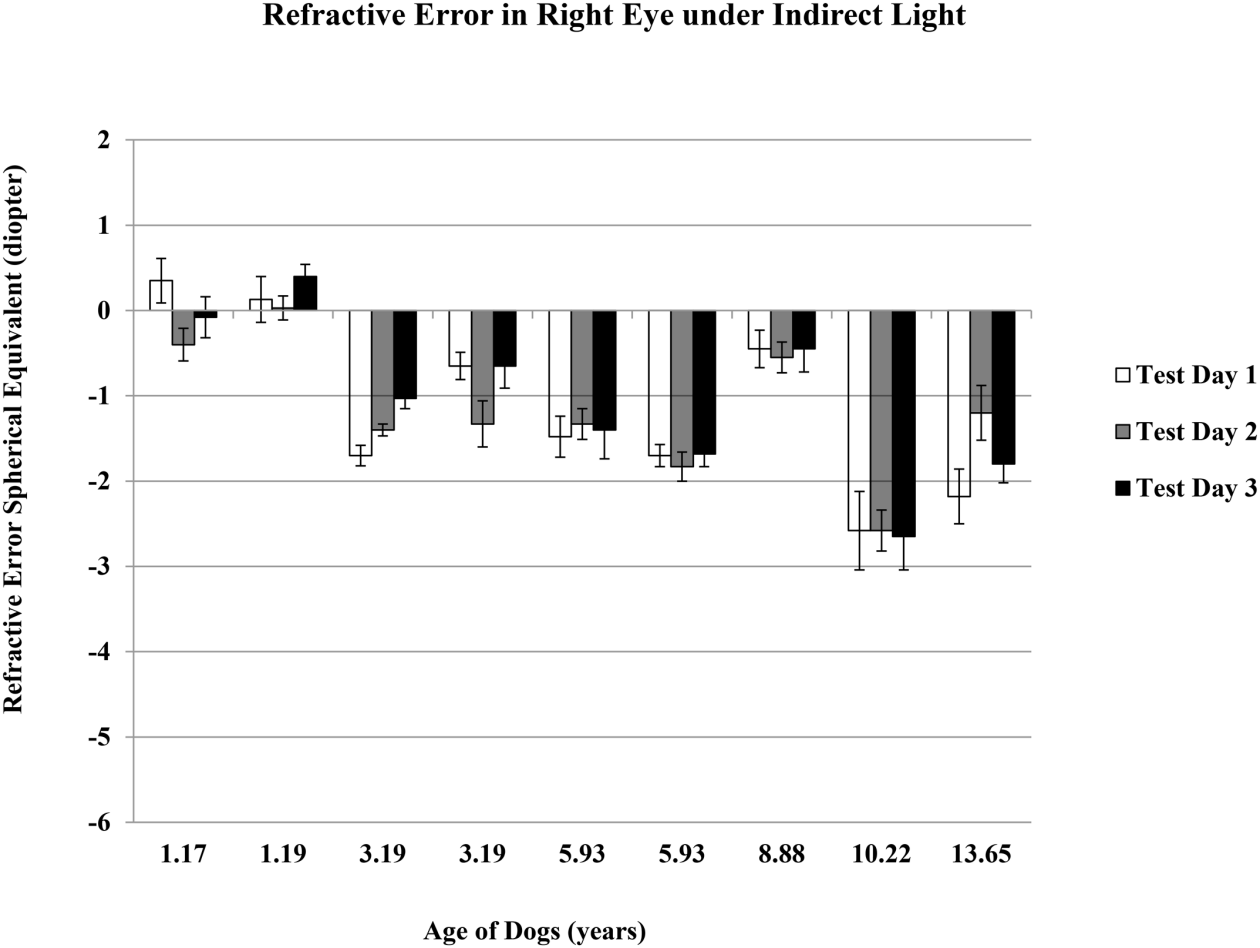
Refractive Error in Right Eyes under Indirect Light. Refractive error spherical equivalent was the average of total five measurements determined using auto-refractor under the indirect light condition of ~ 125 lux. Data was for right eye, and on three test days.

**Fig 4:**
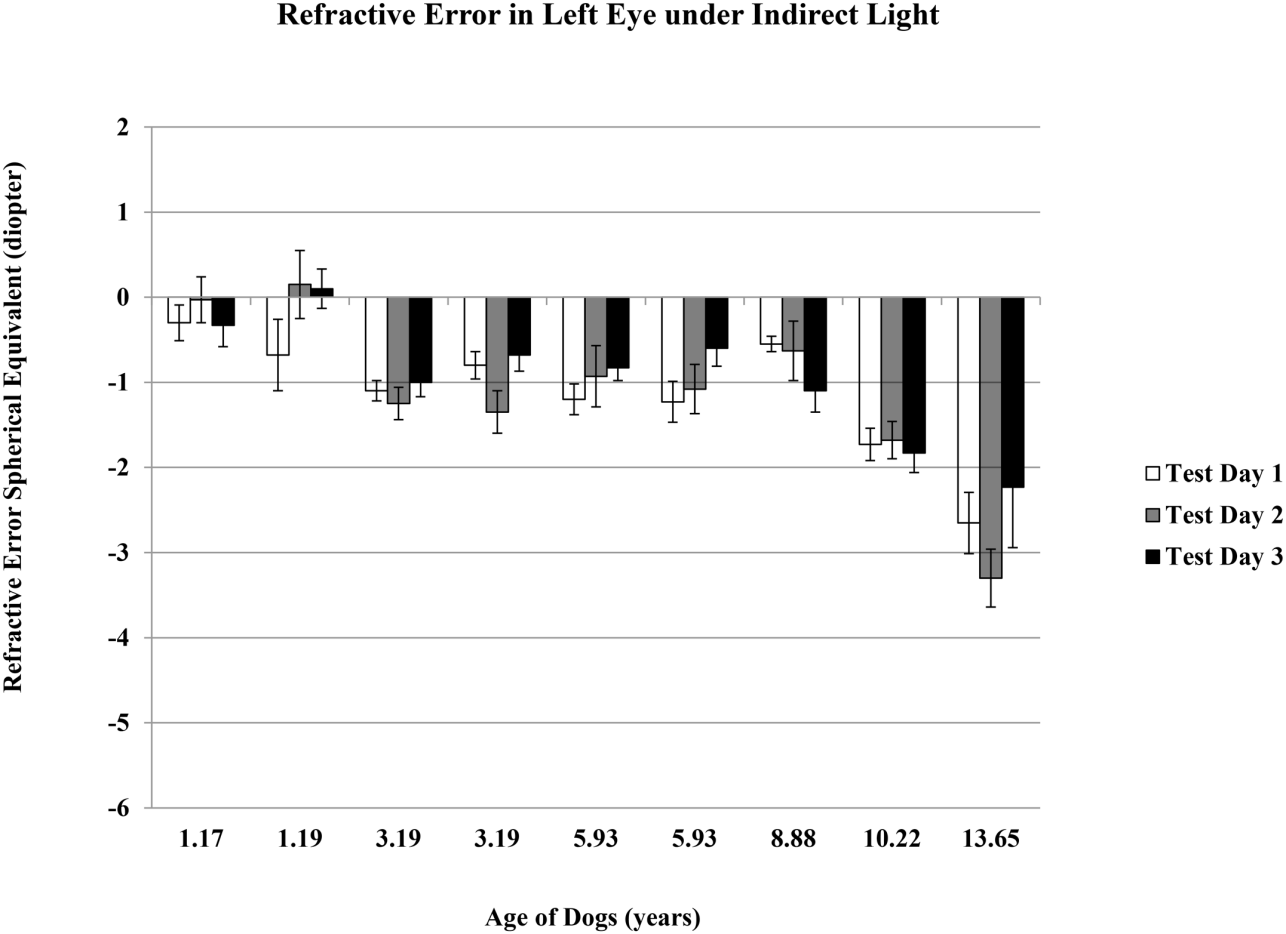
Refractive Error in Left Eyes under Indirect Light. Refractive error spherical equivalent was the average of total five measurements determined using auto-refractor under the indirect light condition of ~ 125 lux. Data was for left eye, and on three test days.

The refractive error mean ± standard error of the nine dogs, ages 1 to 14 years, without cataract (but including two older dogs with moderate and advanced nuclear sclerosis) were -1.61 ± 0.25 D and -1.09 ± 0.28 D under direct and indirect light conditions, respectively. Spherical equivalent refractive error was taken from five measurements per condition, per eye, and was repeated on three different days for each dog within six weeks (Figs 1–4).

There was no difference for either day-to-day or between-eye measurements. The dogs showed a significant myopic shift with aging (average spherical equivalent ranged from plano to refractive error of -3.00 D), suggesting that dogs become more near-sighted as they age (r = -0.48 and -0.73 under direct and indirect light conditions, respectively; p<0.05 for both lighting conditions). Younger dogs were able to make larger accommodation changes from indirect light to direct light conditions, indicating a more flexible lens (r = -0.50, p<0.05).

Additional statistical analyses were done to compare if any differences in spherical equivalent refractive error exist among the first measurement, average of the first three measurements, and average of the five measurements on each day. No statistically significant difference was found in the spherical equivalent refractive error if measured once, three times or five times under different light conditions (Table 2).

**Table 2:** Spherical Equivalent Refractive Error as Measured Once, Three Times or Five Times under Different Light Conditions.

**Spherical Equivalent Refractive Error (Direct light)**	**1st measure**	**Average 3 measure**	**Average 5 measure**
Day 1	-1.47 ± 0.21	-1.70 ± 0.19	-1.78 ± 0.18
Day 2	-1.65 ± 0.40	-1.47 ± 0.26	-1.49 ± 0.26
Day 3	-0.79 ± 0.24	-1.30 ± 0.17	-1.30 ± 0.17
**Spherical Equivalent Refractive Error (Indirect light)**	**1st measure**	**Average 3 measure**	**Average 5 measure**
Day 1	-1.18 ± 0.28	-1.00 ± 0.22	-1.14 ± 0.24
Day 2	-1.11 ± 0.55	-1.20 ± 0.36	-1.12 ± 0.34
Day 3	-1.08 ± 0.25	-0.92 ± 0.29	-0.94 ± 0.24

1^st^ measure: the first measurement of the day

Average 3 measure: the average of the first 3 measurements

Average 5 measure: the average of 5 measurements

Results are presented as mean ± standard error

There was no statistical significant difference comparing three measures on each day: 1st measure, Average 3 measure, or Average 5 measure.

## Conclusions

Our study shows that the clinical handheld portable autorefractor technique used in human pediatrics is applicable to dogs, and is repeatable and sensitive to light conditions.

The mean refractive error in this study group of nine dogs was within the general range of what had been reported among many breeds [9–11]. The refractive error from the four younger dogs in our study were measured to average from -1.00 D and plano in the two ~1 year-old dogs under direct light and indirect light respectively; and average between -1.50 and -1.00 D in the two ~3 year-old dogs under direct light and indirect light conditions, respectively. These refractive error values from plano to -1.50 D are in the range of what has been reported by others [10]. The values from -1.00 to -1.25 D would be equivalent to Snellen 20/50, and -1.75 to -2.00 D would be equivalent to Snellen 20/100.

We observed a significant correlation of refractive error to age under both direct and indirect lights with older dogs being more myopic than younger dogs. In addition, we found that younger dogs were able to make larger accommodation changes from indirect light to direct light conditions, indicating a more flexible lens. These results and correlations are in agreement with previous reports [9–11].

Groth et al [12] compared the Welch Allyn SureSight autorefractor with current standard streak retinoscopy in 50 privately owned dogs (100 eyes) of 20 breeds, free of ocular disease with mean ± SD age of 5.7 ± 3.3 years (range: 6 months–13 years). The refractive error was determined in each eye by two experienced retinoscopists using streak retinoscopy as well as by an autorefractor operated by two different examiners. Measurements were performed before and approximately 30–45 minutes after cycloplegia was induced by cyclopentolate 0.5% and tropicamide 0.5% ophthalmic solutions.

Their study showed that mean ± SD noncyclopleged autorefractor spherical equivalent was −0.42 ± 1.13 D (range: −3.36 to 2.73) D. Mean cyclopleged autorefractor spherical equivalent was 0.10 ± 1.47 D (range: −5.62 to 3.19). Noncyclopleged autorefraction results were not significantly different from streak retinoscopy (whether noncyclopleged or cyclopleged, p = 0.80 and p = 0.26, respectively). The authors concluded that noncyclopleged autorefraction showed good agreement with streak retinoscopy in dogs and can be a useful clinical technique [12].

Based on our evaluation across the varying ages using the same method, we suspect that the varying refractive errors found in previous studies may differ due to many factors such as breed, sample size, gender, age range and whether nuclear sclerosis was present in the study population. For example, some breeds of dogs have a high prevalence of myopia, such as Toy Poodles, English Springer Spaniels and Collies. The cause of the myopia appeared to be mainly from a steeper, more optically powerful crystalline lens, than from excess axial elongation [13]. The elongated vitreous chamber depth was also found to be associated with the increase in myopia in the Labrador Retriever [14]. In this study, we decided to use the same breed for testing the method repeatability and correlating refractive error to age, so that breed would not be a confounding factor.

Based on our observation of the myopic shift in older dogs, we suspect that significant visual alterations may occur in aged dogs similar to that has been reported by human patients who experience nuclear sclerosis. Humans affected with nuclear sclerosis/nuclear cataract report visual disturbances resulting from a myopic shift (from hardening of the lens nucleus), astigmatism, a shift in contrast sensitivity (especially with low-contrast objects), glare, and visual acuity reduction [3]. However, the ability to detect more subtle visual disturbances, especially in the less active older dog, has been limited [3].

**Fig 5:**
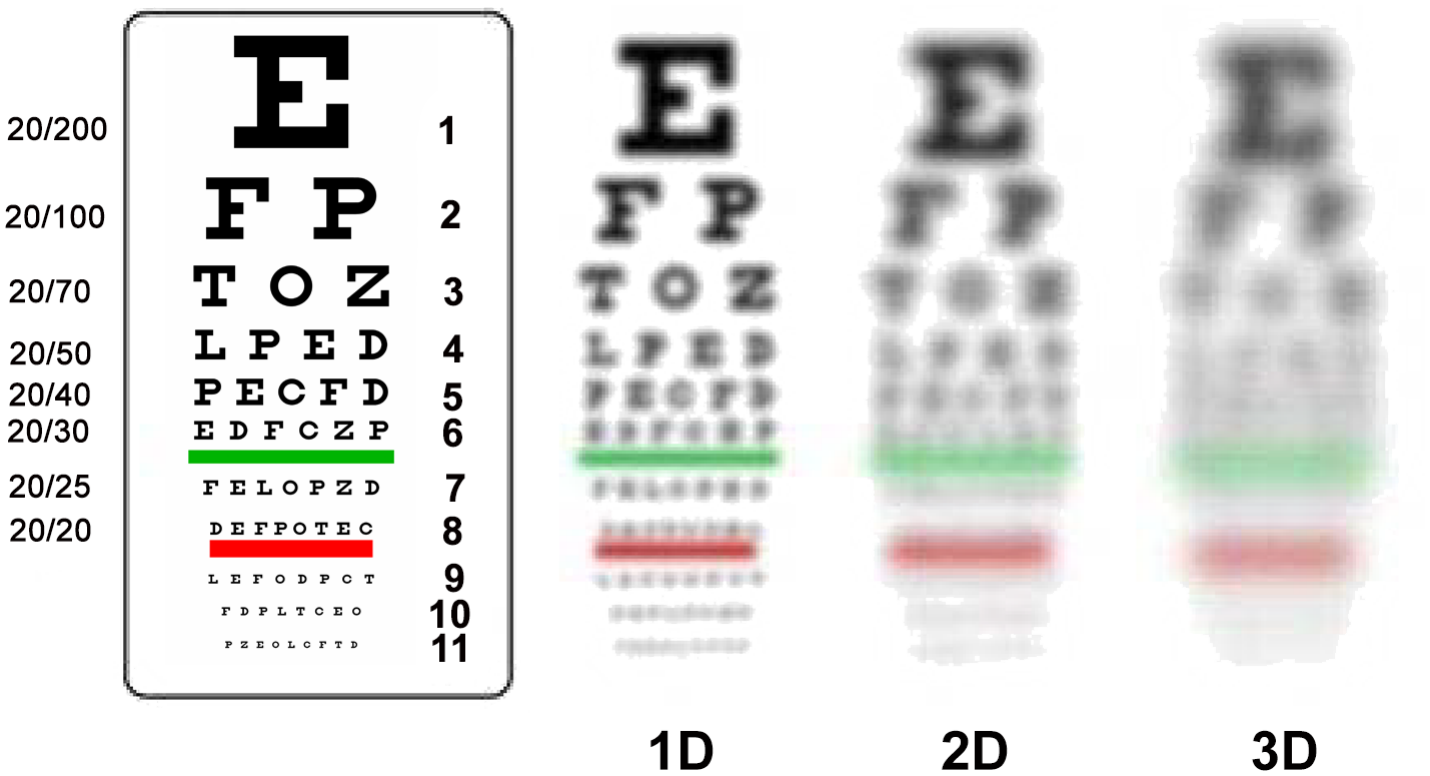
Simulated vision blurriness with different diopter defocus. 1–3 D (diopter) defocus can result in blurry vision simulated based on human subject experience. Similar blurry vision can be expected for dogs that have -1 to -3 D defocus.

The older dogs in our study showed myopic shift close to -2.00 and -3.00 D refractive errors (dogs at age 10.2 and age 13.7 years of age had moderate and advanced nuclear sclerosis without cataracts). Such a degree of myopic shift may result in blurry vision, as simulated in Fig 5, using a typical Snellen vision chart.

The -2 to -3 diopter myopic shift can be a functionally important difference, as observed by Ofri et al [15] in their field training study. Ofri et al [15] studied seven Labrador Retrievers and one Chesapeake Bay Retriever who were trained in field trial competition. Dogs were commanded to retrieve targets at 150 yards. Each dog participated in three trials while their eyes were fitted with plano, +1.50-, or +3.00-diopter (D) contact lenses, applied in random order. Retrieval times were significantly faster with plano lenses than with +1.50- or +3.00-D lenses, but there were no significant differences in times between +1.50- and +3.00-D lenses. The effect of defocus was detected subjectively by professional judges who were unaware of the dogs’ visual acuity, and objectively by measurement of retrieval performance times. Judges blinded to the specific treatment assigned the best performance scores to dogs with plano lenses and the lowest scores to dogs fitted with +3.00-D lenses. The authors concluded that even mild myopic defocusing such as -1.5-D had a significant negative impact on both the subjective and objective assessments of dogs’ performances [15].

Our study further demonstrated that this autorefractor technique is repeatable over separate test days, is sensitive to light conditions reflecting a visual physiological response to environmental changes, and is correlated to age of the dogs. This is technique that a trained technician can easily learn, and use as a clinical measure to understand the visual function of the dog as part of a regular veterinary examination, especially for dog owners for whom the eye condition of the dog is the primary concern for the clinic visit.

The sensitivity and repeatability of the autorefractor to different light conditions that we observed adds more information for future use of this technique in dog eye studies. The greater change of diopters with the change of the light conditions in younger dogs, and an increase in myopic shift associated with age further suggest that this autorefractor method can be used to detect visual function changes in dogs, including vision changes associated with aging.

In conclusion, aging dogs manifest an easily measurable myopic shift. The hand-held autorefractor evaluated in this study can be useful for veterinarians and veterinary ophthalmologists for counseling owners about the age-related vision changes in canine patients. Such autorefractor measurements can be taken by properly trained technicians in a timely and cost efficient manner and can also be used as an objective outcome measure for studying age-related visual function changes in dogs.

## Supporting Information

S1 Appendix Individual Results of Autorefractor Tests. Individual dog refractive error raw data and calculated spherical equivalent were determined using auto-refractor under the indirect light condition of ~ 125 lux and under the direct light condition of ~ 1,100 lux. The indirect lighting condition (illumination ~125 lux) was set with indoor light from an adjacent room coming through an open door into a dark room with dogs facing the incoming light. The direct light condition (illumination ~1,100 lux) was set in the same position but with the light turned on in the examination room. Eyes were first tested under the indirect lighting condition, followed by direct lighting at the same setting. Dataset included results for nine dogs under two light conditions for both left and right eyes, five measurements per eye per condition, and for three different test days within six-week time period.
(XLSX)
